# Plasma proteomics reveals coagulation, inflammation, and metabolic shifts in H-type hypertension patients with and without acute ischemic stroke

**DOI:** 10.18632/oncotarget.22233

**Published:** 2017-11-01

**Authors:** Feng Zhou, Lv Zhou, Tie Guo, Nianzhen Wang, Haizhen Hao, Yanhui Zhou, Dan Yu

**Affiliations:** ^1^Department of Neurology, Affiliated Haikou Hospital at Xiangya Medical School of Central South University, Haikou 570208, Hainan, China

**Keywords:** acute ischemic stroke, differentially expressed proteins, H-type hypertension, iTRAQ, plasma proteomics

## Abstract

Systematic profiling of a larger portion of circulating plasma proteome provide opportunities for unbiased discovery of novel markers to improve diagnostic, therapeutic, or predictive accuracy. This study aimed to identify differentially expressed proteins (DEPs) in plasma that could provide overall insight into the molecular changes of both H- type hypertension (HH) and HH-related acute ischemic stroke (AIS). This study used an iTRAQ-based LC-MS/MS proteomics approach to screen for plasma DEPs in HH patients with and without AIS, and controls. After excluding highly abundant plasma proteins, more than 600 proteins, and their relative levels, were identified. Of these, 26 DEPs, each showing > 1.2-fold change, were identified in HH and HH-related AIS patients compared with controls. Bioinformatics analysis revealed that these DEPs were enriched in 21 functional gene ontology items; “blood coagulation” was the most predominant pathway showing enrichment. Of these, eight DEPs were located in the hub position of networks involved with protein-protein interactions. AT-3, CRP, ApoB, and AHSG were further validated in each group by enzyme-linked immune sorbent assays. Comparing HH-related AIS with HH, the areas under the curve for AT-3, CRP, ApoB, and AHSG were 0.698, 0.892, 0.626, and 0.847, respectively. This proteomic profiling study provided enhanced pathophysiological understanding of the regulatory processes involved in coagulation, inflammation, and metabolism, and identified a panel of novel biomarkers for detecting HH-related AIS during its pre-stroke stage.

## INTRODUCTION

Stroke, a multifactorial disease, is one of the leading health issues with permanent physical and neurological disabilities worldwide, and results in high morbidity and mortality rates [1, 2]. Of all strokes, 85% of cases involve ischemic stroke [1]. Despite progress in acute treatment protocols for ischemic stroke, prevention remains the most effective approach to reduce impact upon personal and public health [3]. The prevalence and incidence of ischemic stroke has significantly increased with the increasing prevalence of H- type hypertension (HH), especially in patients without folate supplement [4]. Interactions between inflammation, immune reaction, and oxidative damage are responsible for the development of atherosclerotic ischemic stroke by hyperhomocysteinemia [5-7], but the underlying pathophysiological pathways leading from HH to acute ischemic stroke (AIS) remain poorly understood. More potent biomarkers are needed for the detection of HH-related AIS during its pre-stroke stage when it can be intervened more effectively.

Protein biomarkers that reflect the molecular states of specific diseases are central to the clinical decision making process [8]. Nevertheless, studies of human stroke are often limited because brain tissue and cerebrospinal fluid cannot be collected easily from living patients. As damage to the blood-brain barrier is one of the major consequences of AIS, proteins associated with the pathogenesis of stroke may appear in the plasma. Consequently, the plasma proteome holds vital pathophysiological information, both pre- and post- stroke, and is thus considered as a valuable window for assessing pathophysiological features [9].

Proteomics represents a powerful comprehensive analysis tool which allows us to compare the overall protein status of various samples in a biological system with a specific pathological condition [10]. The additional use of isolated tags for relative and absolute quantitation (iTRAQ) is a sensitive profile that uses a single mass spectrometry analysis to identify clinically relevant less abundant proteins in plasma [11]. Several previous studies have investigated the proteomics profiles of stroke and have provided key information relating to the pathophysiological features of stroke [12, 13]. Until now, however, proteomics has not been used to investigate differently expressed proteins (DEPs) in the plasma of HH patients with and without AIS.

In the present study, we aimed to screen potential candidate markers in HH, HH- related AIS and controls using an iTRAQ labeling approach coupled with liquid chromatography and tandem mass spectrometry (LC-MS/MS). We then validated the experimental findings of our discovery phase using enzyme-linked immune sorbent assays (ELISA).

## RESULTS

### Clinical profiles of subjects

All subjects were ethnically Han Chinese recruited from Haikou city in Hainan province, China. The three groups of patients were age- and sex- matched; demographic characteristics and clinical information are presented in Table 1. There were no statistical differences between the HH group and HH-related AIS group in terms of age, sex, body mass index, smoking, family history of stroke, hypertension, diabetes, hyperlipidemia, or the use of medication (*p* >0.05).

**Table 1:** Demographics and clinical characteristics of participants

Variable	HH-related AIS (n =30)	HH (n =30)	Controls (n =30)
Age, years	69.67±13.65	71.42±8.39	65.36±12.58
Female sex, n (%)	18(60.0%)	19(63.3%)	17(56.7%)
Smoker, n (%)	5(16.7%)	4(13.3%)	6(20.0%)
Homocysteine, μmol/L	18.62±2.21^*^	19.06±3.47^*^	6.41±2.02
Hypertension, n (%)	25(83.3%)	24(80.0%)	N/A
Diabetes, n (%)	6(20.0%)	N/A	N/A
Hyperlipidemia, n (%)	12(40.0%)	11(36.7%)	12(40.0%)
Anti-hypertensive, n (%)	23(76.7%)	21(70.0%)	N/A
Anti-diabetic, n (%)	20(66.7%)	N/A	N/A
Statin therapy, n (%)	6(20.0%)	N/A	N/A
Family history of IS, n (%)	14(46.7%)^*^	14(46.7%)^*^	10(33.3%)

N/A: not applicable.

All values are presented as N (%), Mean ± standard deviation, properly.

Compared with controls, ^*^p <0.05.

### Highly abundant proteins depletion

Highly abundant proteins were depleted prior to proteomics analysis. This allows the detection of low abundance proteins with much less interference. Protein normalization was confirmed using 12% SDS-PAGE (Supplementary Figure 1).

### Identifying and quantifying peptides and proteins

**Figure 1:**
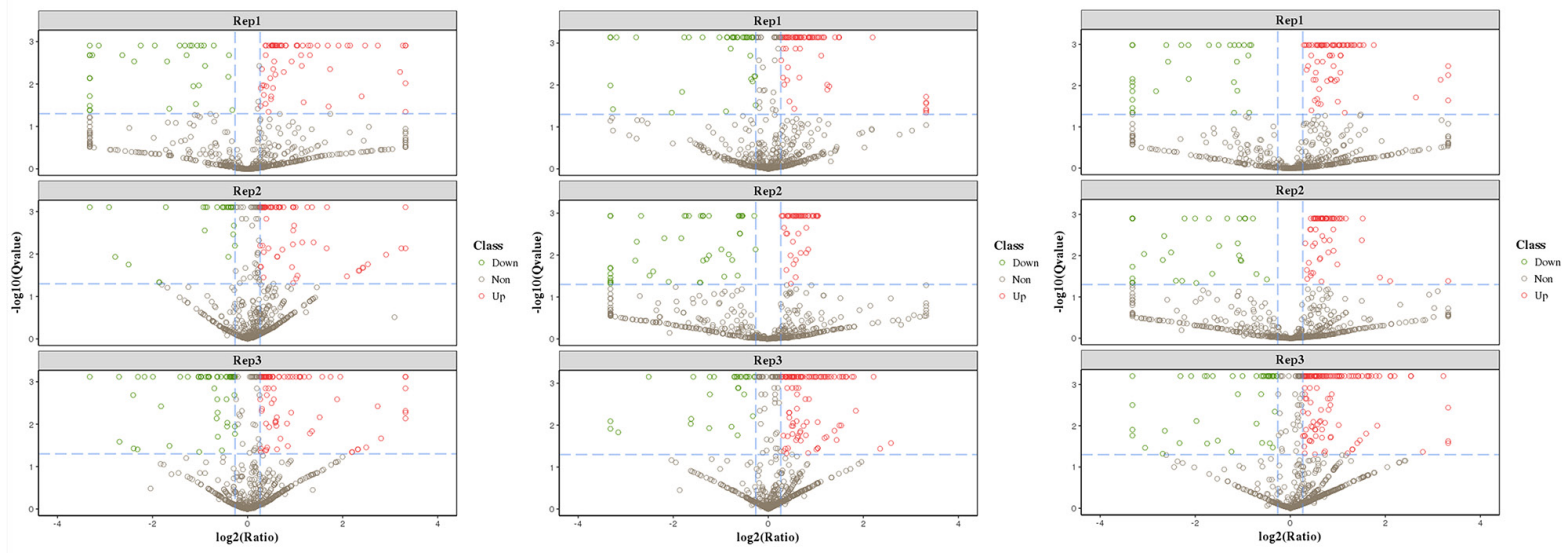
Volcano plots of differentially expressed proteins in HH-related AIS patients *versus* healthy controls **(A)**, HH patients *versus* controls **(B)**, and HH-related AIS *versus* HH patients **(C)**. Red indicate the up-regulation and green indicate the down-regulation of differentially expressed proteins >1.2-fold.

**Figure 2:**
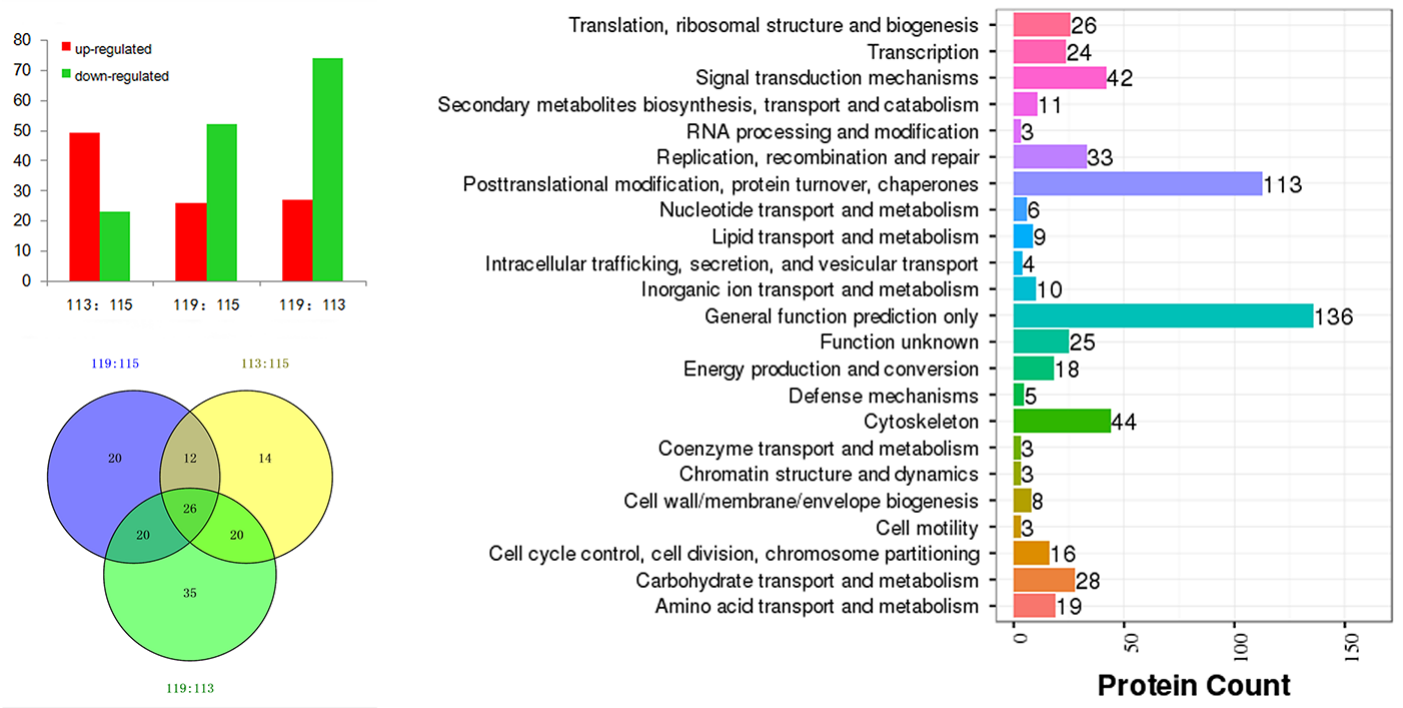
Differentially expressed proteins in HH-related AIS patients *versus* healthy controls (119:115), HH patients *versus* controls (113:115), and HH-related AIS *versus* HH patients (119:113) **(A)**; COG analysis **(B)** and Venn chart **(C)**.

**Figure 3:**
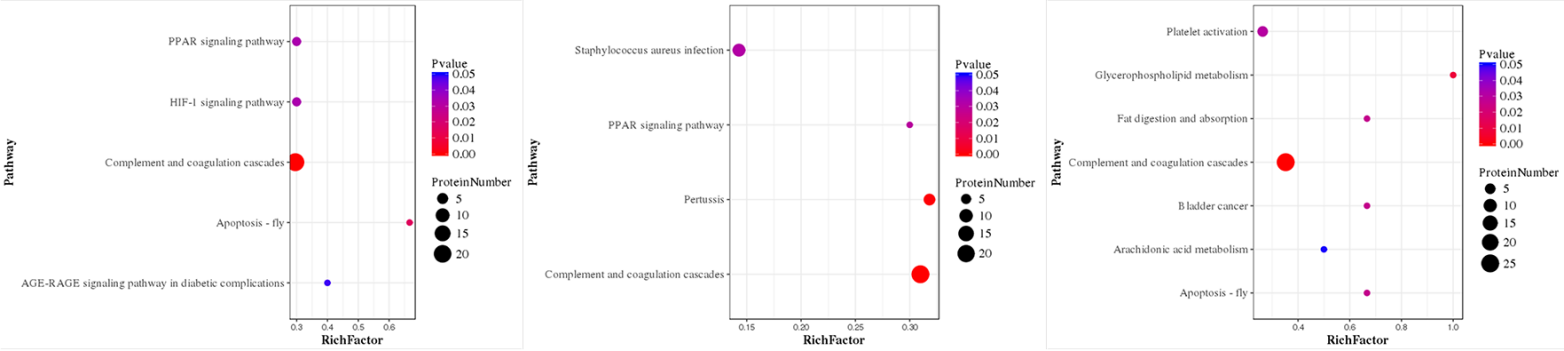
Pathway-Enrichment analysis of HH-related AIS patients *versus* healthy controls **(A)**, HH patients *versus* healthy controls **(B)**, and HH-related AIS *versus* HH patients **(C)**. P-value for the “component and coagulation cascades pathway” was 7.371119e-10, 4.66942e-10, and 5.61542e-10, respectively.

The iTRAQ-based quantitative proteomics analysis of HH, HH-related AIS, and controls using LC-MS/MS revealed the identity of 4705 distinct peptides with a false discovery rate of < 1% and p < 0.05 (Supplementary Figure 2). The results from three biological replicates in LC-MS/MS analysis of 611, 604 and 692 proteins were observed, respectively. Volcano plots showing protein ratios of HH *versus* Controls, and HH- related AIS *versus* Controls, are represented in Figure 1. A significant increase (> 1.2-fold change) of 26 DEPs was observed in HH- related AIS and 49 DEPs in HH, whereas 52 and 23 DEPs (< 0.83-fold change) were significantly reduced in HH- related AIS and HH compared to controls, respectively. In total, 27 DEPs were up-regulated, and 74 DEPs were down-regulated in HH- related AIS compared to HH (Figure 2A). The key item arising from COG (cluster of orthologous groups of proteins) analysis was “general function prediction”, followed by “posttranslational modification, protein turnover, chaperones” and “cytoskeleton”. The results of COG analyses are shown in Figure 2B. Pathway- enrichment analyses are shown in Figure 3; “complement and coagulation cascades” was the most importance item identified.

### Twenty-six DEPs were associated with both HH and HH-related AIS

In total, 26 DEPs were identified when investigating the HH group *versus* controls, HH- related AIS group *versus* controls, and HH- related AIS group *versus* HH group (Figure 2C). Detailed lists of these DEPs, along with ratio information, is shown in Table 2.

**Table 2:** The identification of 26 differentially expressed proteins

Accession number	Gene names	Protein names	Mass (Da)	Unique peptides	113:115	119:115
P02741	CRP	C-reactive protein	25175.73	6	2.027	1.972
P02765	AHSG	Alpha-2-HS-glycoprotein	40080	10	1.244	3.876
P32119	PRDX2	Peroxiredoxin-2	22031.29	7	1.656	1.314
P05546	SERPIND1	Heparin cofactor 2	57187.26	20	1.605	1.808
P04114	APOB	Apolipoprotein B-100	516633.4	24	2.002	1.441
P01871	IGHM	Immunoglobulin mu heavy chain	64226.46	1	1.61	2.72
P02655	APOC2	Apolipoprotein C-II	11258.74	5	1.364	2.083
P03952	KLKB1	Plasma kallikrein	73414.63	19	1.369	2.037
P0DJI8	SAA1	Serum amyloid A-1 protein	13562.53	3	1.604	1.205
P08253	MMP2	72kDa type IV collagenase	74900.11	10	1.240	0.767
P02788	LTF	Lactotransferrin	79995.61	15	1.475	0.695
O15360	FANCA	Fanconi anemia group A protein	164820.2	1	0.491	2.315
P02679	FGG	Fibrinogen gamma chain	52088.09	25	0.599	1.280
P43652	AFAM	Afamin	70944.73	13	1.363	0.718
P00738	HP	Haptoglobin	45842.81	9	1.910	0.811
P02790	HPX	Hemopexin	52366.54	13	1.292	0.697
P04180	LCAT	Phosphatidylcholine-sterol acyltransferase	49870.21	7	1.586	0.787
Q9NP78	ABCB9	ATP-binding cassette sub-family B member 9	84859.21	1	0.301	1.276
P36955	SEPRINF1	Pigment epithelium-derived factor	46436.36	10	1.487	0.737
P19823	ITIH2	Inter-alpha-trypsin inhibitor heavy chain H2	106834.8	25	0.737	0.471
P01008	SERPINC1	Antithrombin-III	53007.03	20	0.757	0.556
Q02878	RPL6	60S ribosomal protein L6	32746.64	1	0.757	0.585
Q96KN2	CNDP1	Beta-Ala-His dipeptidase	56766.15	19	0.818	0.698
P02766	TTR	Transthyretin	15973.08	7	0.643	0.271
P68871	HBB	Hemoglobin subunit beta	16084.32	8	0.675	0.419
P02787	TF	serotransferrin	79276.48	25	0.421	0.518

113:115, the HH group *versus* the healthy control group; 119:115, the HH-related AIS group *versus* the healthy control group.

**Figure 4:**
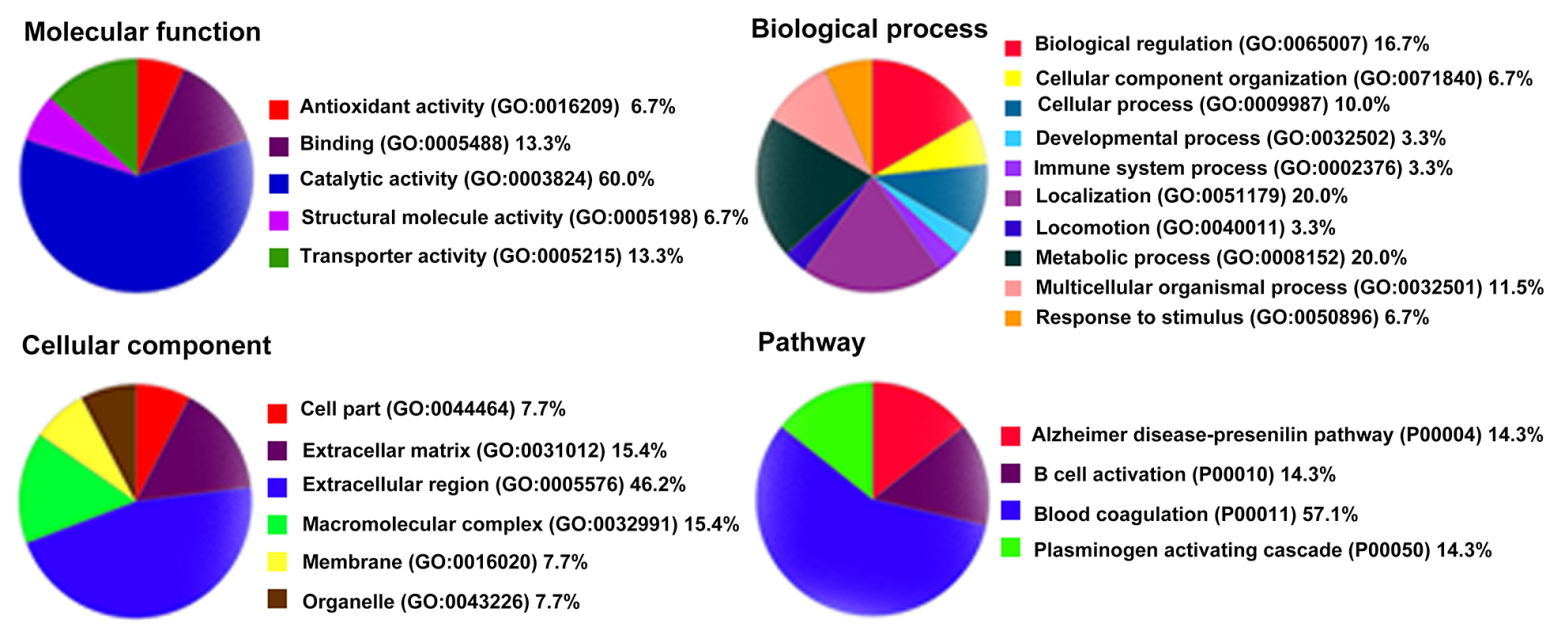
Gene ontology analysis of the 26 differentially expressed proteins by PANTHER

**Figure 5:**
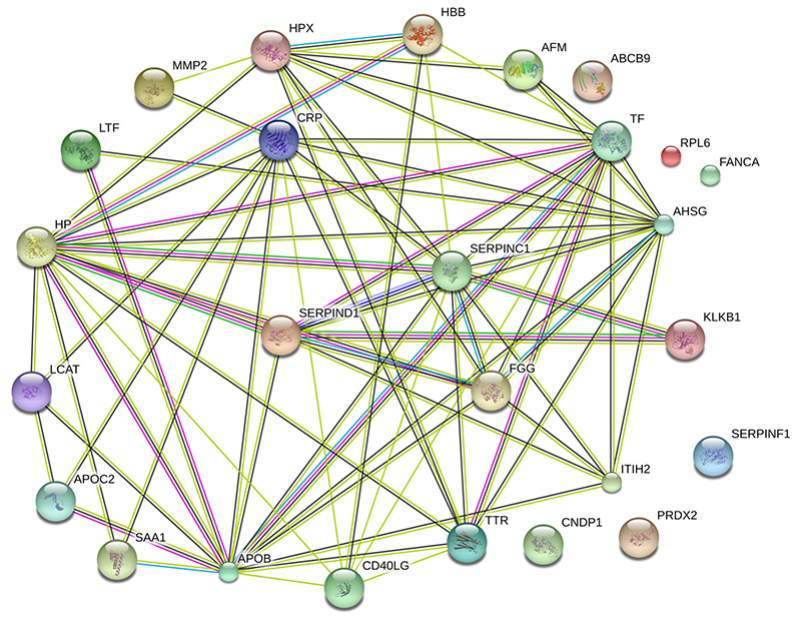
Protein-protein interactions between the 26 DEPs by STRING Minimum required interaction score: 0.4; average node degree: 5.62; average local clustering coefficient: 0.567; Protein-protein interactions enrichment p-value: <0.001. AT-3, CRP, ApoB, and AHSG were found to be located at hub positions within networks of protein-protein interactions.

Panther (http://www.pantherdb.org) was used to perform gene ontology (GO) analysis. As shown in Figure 4, the biological process category of GO analysis shortlisted “metabolic process”, “localization”, and “biological regulation” as some of the most perturbed processes, whereas “extracellular region” was the key cellular component that was enriched in the perturbed proteome. Significantly enriched molecular function, including “catalytic activity”, “binding”, and “transporter activity” were also shortlisted and are shown in Figure 4. Pathway analysis also shown that “blood coagulation” was significantly over-represented (Figure 4). The DEPs included antithrombin-III (AT-3), fibrinogen gamma chain, heparin cofactor 2, and plasma kallikrein. Moreover, String (http://string-db.org) shown that AT-3, fibrinogen gamma chain, heparin cofactor 2, C-reactive protein (CRP), transthyretin, apolipoprotein B (ApoB), alpha-2-HS-glycoprotein (AHSG) and haptoglobin were located at the hub position of networks for protein-protein interactions (Figure 5). Collectively, bioinformatics analysis identified plasma DEPs in both HH and HH-related AIS patients which are involved in essential pathophysiological pathways of blood coagulation cascade, inflammation, and metabolic processing.

### ELISA validation

**Figure 6:**
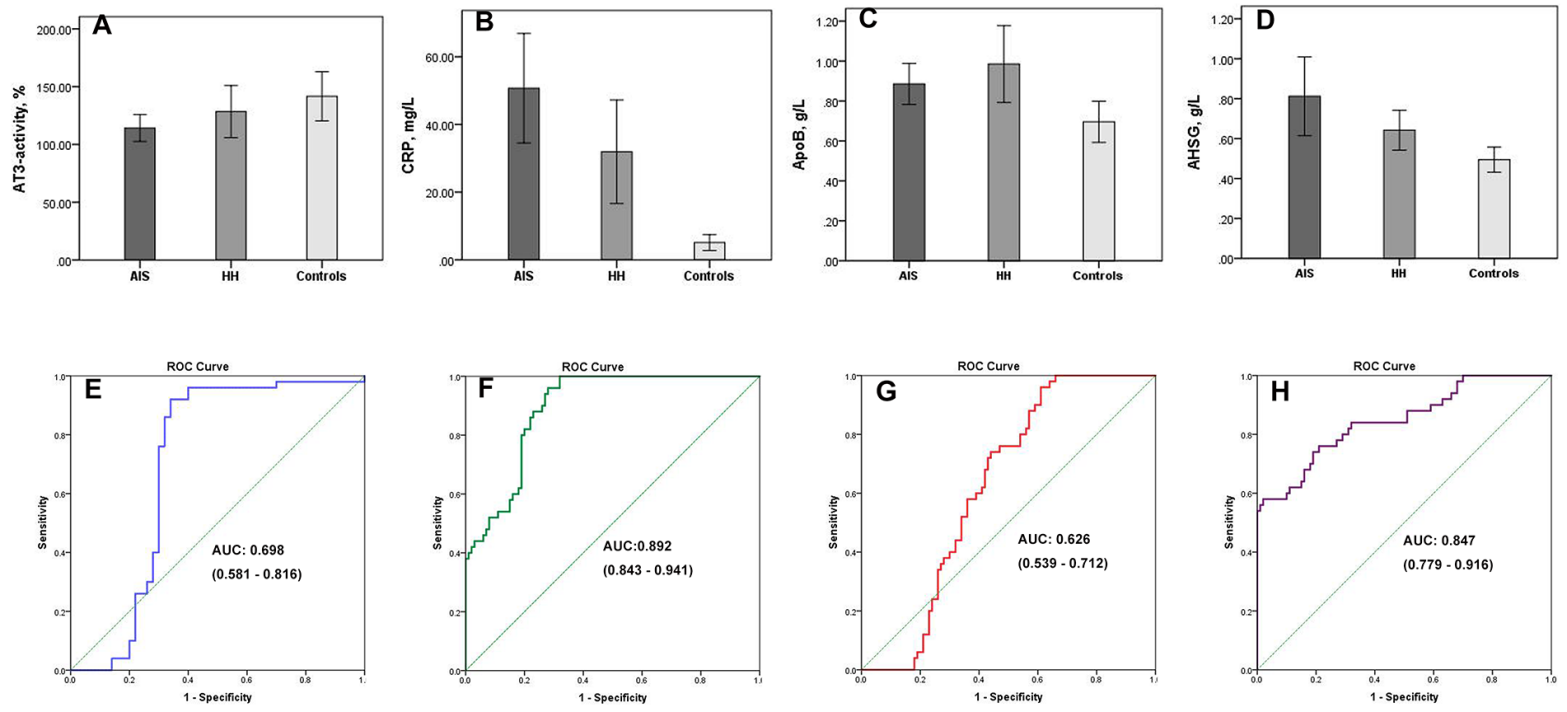
Four differentially expressed proteins validated by ELISA assays Plasma levels of AT-3 **(A)**, CRP **(B)**, ApoB **(C)**, AHSG **(D)** are shown in HH-related AIS patients, HH patients, and healthy controls, respectively. Compared with controls, ^*^p <0.05. Receiver operating characteristic curves and areas under the curve (AUC) of AT-3 **(E)**, CRP **(F)**, ApoB **(G)**, AHSG **(H)** were used to differentiate the HH-related AIS group from the HH group; AUC of AT-3, CRP, ApoB, and AHSG were 0.698, 0.892, 0.626, and 0.847, respectively.

To verify the results of the LC-MS/MS experiment, ELISA assays were used to quantify the expression of AT-3, CRP, ApoB, and AHSG in the plasma. Compared to controls, both HH- related AIS and HH patients were found to have lower plasma levels of AT-3. CRP, ApoB, and AHSG, all exhibited higher levels in HH and HH- related AIS patients than in controls (*p* < 0.05, Figure 6A-6D). These data are consistent with the changes in expression revealed by our proteomics approach. Comparing HH- related AIS with HH, the areas under the curve for AT-3, CRP, ApoB, and AHSG were 0.698, 0.892, 0.626, and 0.847, respectively (Figure 6E-6H). Therefore, it can be concluded that these specific DEPs are implicated in the exacerbation of pathophysiological changes in HH- related AIS patients and can imply a deleterious change from the HH condition.

## DISCUSSION

Using an iTRAQ-based LC-MS/MS approach, we identified a series of surrogate plasma biomarkers which correlated with the pathophysiological changes of both HH and HH- related AIS. To the best of our knowledge, this clinical study is the first to identify DEPs in HH and HH- related AIS patients compared with healthy controls. A total of 26 DEPs were excreted in the plasma of HH patients with and without AIS. Accordingly, GO analysis identified proteins enriched in 21 functional GO items. The top enriched item in each category was “extracellular region” in the cellular component, “catalytic activity” in the molecular function, “metabolic process” in the biological process, respectively. “Blood coagulation” was identified as the most important pathway. Finally, AT-3, CRP, ApoB, and AHSG levels were validated using ELISA. Analysis suggested that the combination of these validated DEPs could represent useful plasma biomarkers for HH and HH-related AIS.

### AT-3 and the coagulation cascades

HH and HH- related AIS are both associated with coagulopathy. Hypercoagulable states are characterized by abnormally high circulating levels of thrombin. *Ex vivo* subjects with hyperhomocysteinemia have been linked with hypercoagulable states, which has been proposed as a predisposing factor for the development of atherosclerotic cerebral ischemia [14]. It is documented that S- or N-homocysteinylation of proteins can induce cellular toxicity and endothelial injury, activated adaptive immune responses, and enhance thrombosis caused by N-homocysteine-fibrinogen [15, 16]. Furthermore, hyperhomocysteinemia triggers a series of mechanisms (activation of factor XII, inhibition of protein C activation) which are potentially responsible for the disrupted activation of the coagulation system and an impairment of antithrombotic mechanisms which can interfere with the fibrinolytic system, and result in a hypercoagulable state [17, 18]. Furthermore, the activation of platelets induced by hyperhomocysteinemia is known to facilitate the formation of thrombus [17, 19]. AT-3, which is predominantly synthesized by the liver, play a protective role in the coagulation response as a main inhibitor of thrombin and factor Xα [20]. Inhibition of thrombin by AT-3 is probably the principal mechanism responsible for the removal of thrombin produced during thrombosis [21]. When ischemic stroke occurs, a significant quantity of thrombin is activated, the consumption of AT-3 immediately increases, and subsequently, the level of plasma AT-3 decreases rapidly.

AT-3 appears to be comparably less affected, and is primarily altered by mutations, consumption or hepatocellular dysfunction [22]. Familial AT-3 deficiency is an autosomal dominant disorder with a typical onset at 10-25 years old [23]. The subjects in the present study were more than 40 years old without family history of early onset of thrombosis. Therefore, this genetic disorder was rejected. The aberrant synthesis, or reduced release, of AT-3 is usually caused by dysfunction of the blood vessel endothelium and liver disease. Accordingly, patients with vessel, heart and liver disease were excluded. Our findings concur with prior observations that plasma AT-3 levels are significantly reduced in HH and HH-related AIS patients [24, 25]. In brief, AT-3, represents a useful molecule linked to HH and HH- related AIS pathobiology.

### CRP and inflammation

It has been well recognized that homocysteine induced vascular inflammation plays a critical role in the pathophysiology of HH [26, 27, 28] and atherosclerosis [29]. Of note, some pro-inflammatory proteins, including CRP and serum amyloid A-1 protein, act on endothelial cells resulting in acute effects of thrombosis and chronic effects of atherosclerosis [30], subsequently increasing the risk of stroke. Combined evidence suggests that the high-risk stroke population with CRP levels in the highest quartile are 2 to 7-fold more likely to develop stroke than those with CRP levels in the lowest quartile [29]. Previous meta-analyses [31, 32] suggested that elevated plasma CRP concentration is also positively associated with ischemic stroke risk. Furthermore, high CRP levels in the acute phase of ischemic stroke can predict adverse outcomes [33]. In addition, genetic studies have indicated a significantly association between *CRP* gene mutation and the risk of ischemic stroke.

Variation in the observed associations between CRP levels and the risk of incident stroke has been attributed to differences in patient demographics, the prevalence of comorbid conditions, genetics, and other environmental factors [29]. Thus, in the present study, subjects were enrolled using strict exclusion criteria. Comparing the HH- related AIS group with the HH group, the AUC for CRP was 0.892, thus indicating that CRP represents a good biomarker. Since CRP is a commonly measured biomarker, we believe that it could be a valuable contributor to a future multi-marker panel for determining the risk of stroke in HH patients.

### Apolipoprotein B-100, AHSG and the metabolic process

Apolipoprotein (Apo) B-100, the dominant form of ApoB, is synthesized in the liver. In most conditions, more than 90% of ApoB-100 in the blood is found in low-density lipoprotein cholesterol [34]. Although ApoB is pivotal for lipid absorption and triglyceride homeostasis, high levels in the plasma can induce atherosclerosis [35]. Hyperhomocysteinemia has also been shown to enhance the production of cholesterol and promote the secretion of ApoB [36], and thus worsen atherosclerosis. The stimulatory effect on cholesterol synthesis is mediated via the enhancement of HMG-CoA reductase, a critical protein in cholesterol biosynthesis. This plausible mechanism could explain the observed relationship between hyperhomocysteinemia and the development of atherogenesis and stroke. ApoB level has been shown to be significantly higher in stroke patients compared to controls [37]. Furthermore, *APOB* gene mutations can have profound influence on the levels of ApoB protein. Four SNPs of *APOB* gene significantly associated with the risk of ischemic stroke [38]. The findings of the current study concur with previous studies, in that patients with HH or HH-related AIS, show an increased level of ApoB.

AHSG, a plasma glycoprotein secreted predominantly by the liver, has been associated with atherosclerotic arterial calcifications [39], insulin resistance [40], cardiovascular disease [41], and particularly, ischemic stroke [42, 43]. These associations may suggest an indirect role of AHSG in the pathogenesis of ischemic stroke through its influence upon stroke risk factors. A prospective cohort study, the EPIC-Potsdam study, shown that individuals in the highest quintile of AHSG had significantly increased ischemic stroke risk (highest *versus* lowest quintile: odds ratio = 3.93, 95% confidence interval: 2.17-7.12.) [42]. Another nested case-control study shown that circulating AHSG was higher in women with an increased body mass index and total cholesterol level at baseline; however, AHSG quartiles were not significantly associated with ischemic stroke risk [43]. The inconsistencies between the findings of previous studies may be due to differences in population characteristics, the prevalence of comorbidity, and variations in endpoints. Additionally, elevated AHSG levels and the *AHSG* gene rs4917 polymorphism were associated with ischemic stroke risk [44]. Higher AHSG levels, in both HH and HH- related AIS, compared to controls, is in agreement with a metabolic disorder scenario in this clinical context. These mechanisms warrant further evaluation to clarify whether the high circulating level of AHSG is a consequence of HH or a predisposing factor for subsequent stroke events.

There are some limitations to consider. First, the fact that our study focused upon the Han Chinese population, limits the generalizability of our findings to other ethnicities. Second, anticoagulants can influence proteomics investigations by affecting the plasma proteome [45]. However, plasma shows more stability than serum in terms of not releasing peptides from blood clots [46]. Furthermore, proteomics studies have reported that EDTA-coagulated plasma samples yielded the most reproducible results compared to other anticoagulant reagents [47]. Moreover, human blood is easily obtained, and is of significant clinical importance, in comparison to other biological samples. Therefore, we chose plasma rather than serum as the sample source. Third, Patients in the HH group and the HH-related AIS group, were taking antihypertensive therapies, types of antihypertensive drugs are metabolized through the liver. In spite of the fact that we ruled out subjects with abnormal overall liver function upon admission, the relative effects of anti-hypertensive drugs and other drugs (e.g.: anti-diabetic, statin), on DEPs remain unknown. Finally, further research on the mechanisms underlying the expression and action of the DEPs involved in HH and HH-related AIS is now required.

In summary, this study analyzed the plasma proteomics profiles of HH and HH- related AIS. Results obtained from this comprehensive proteomics analysis revealed changes in coagulation, inflammation, and metabolic process as the possible cues contributing towards HH and HH- related AIS. We speculate that apart from parameters associated with blood pressure and hyperhomocysteinemia, the validated proteins identified by this study represent important molecular markers for the pathophysiological features of HH and HH- related AIS. Collectively, these findings are likely to assist future research seeking to develop novel ways to protect patients against HH-related stroke.

## MATERIALS AND METHODS

### Ethics

This study protocol was approved by the Human Research Ethics Committee of Haikou Municipal Hospital and was conducted in accordance with the Declaration of Helsinki. All participants, or their legally authorized representatives signed the consent form.

### Design protocol and diagnosis criteria

**Figure 7:**
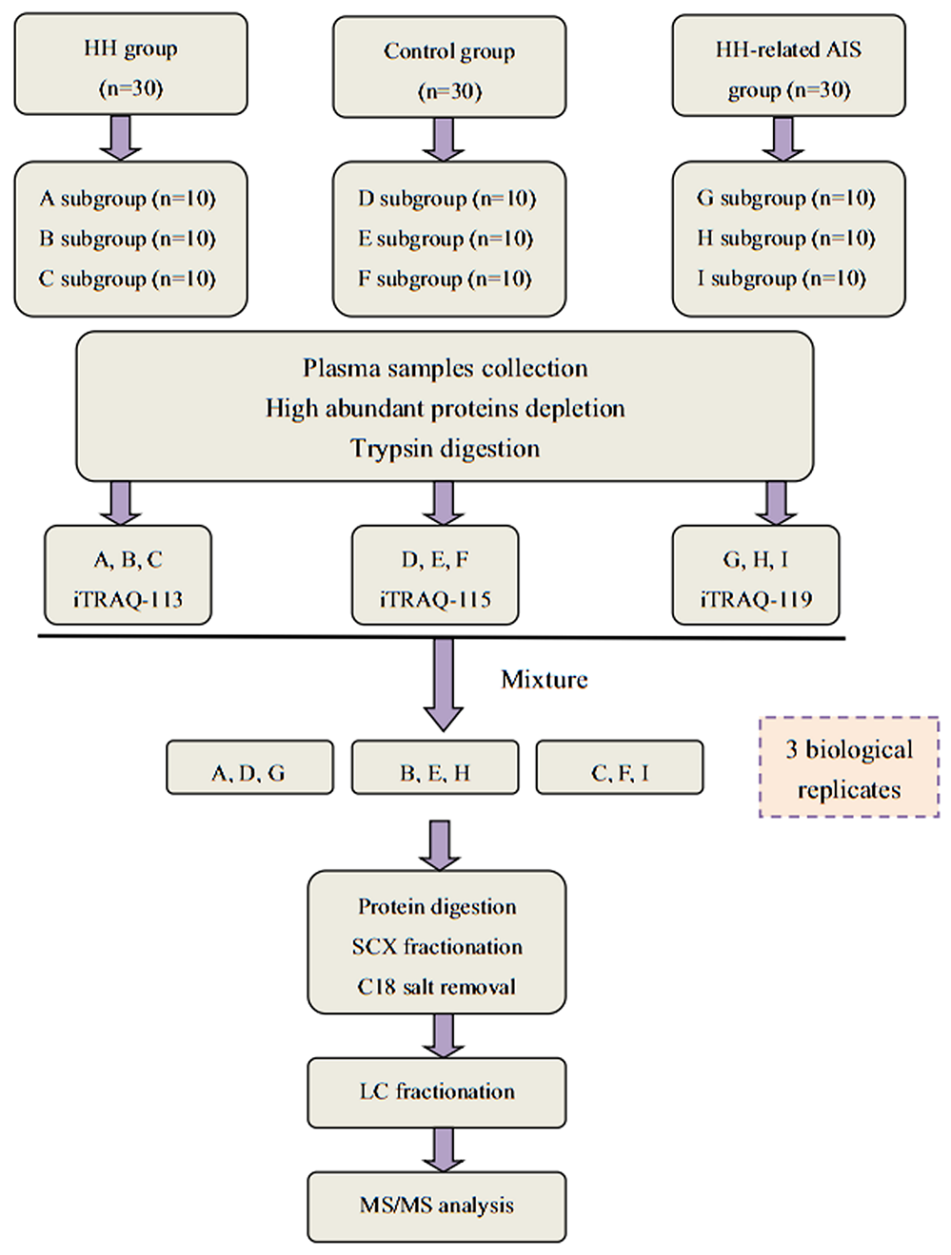
A flow chart showing our iTRAQ-based LC-MS/MS proteomics approach

A schematic representation of the study workflow is shown in Figure 7. All donors were enrolled in Haikou Municipal Hospital between August 2016 and March 2017. All subjects were divided into 3 groups: HH- related AIS, HH, and controls. Thirty HH- related AIS patients were recruited from the emergency room or neurology department within 24hrs of the incident from stroke onset. We also recruited 30 HH patients without other basic disease, and 30 healthy control subjects. The controls had all undergone evaluation in our physical examination center, and were not taking any drugs except for anti-hypertensive medication and/or B vitamins within the previous 2 weeks. An additional 20 individuals were enrolled into each group for the ELISA.

The diagnosis of ischemic stroke was made following the criteria of the World Health Organization [48]. The HH- related AIS patients (diagnosed with brain computed tomography or magnetic resonance imaging) were all classified as the large-artery atherosclerosis subtype in accordance with TOAST classification [49]. The severity of stroke was assessed by NIHSS on admission (scores ranged from 8 to 14). HH was defined as when SBP ≥140mmHg and/or DBP ≥90mmHg or when patients were receiving current anti-hypertensive therapy, in combination with plasma homocysteine >15μmol/L. Patients were excluded in accordance with the following criteria: 1) age <40 years; 2) transient ischemic attack, recurrent stroke, and hemorrhagic stroke; 3) traumatic brain injury, brain tumor; 4) infection; lung, heart, liver or renal disease and complications thereof. Demographic and clinical characteristics were collected on admission (Table 1).

### Plasma sample preparation

Blood samples were collected from HH- related AIS patients before the administration of medication. Blood samples from HH and controls were collected in the morning following overnight fasting. Blood (3ml) was collected in K2 EDTA-lined vacuum tubes (Medray Inc., Shenzhen, China) and plasma was prepared via centrifugation at 3000*g* and 4°C for 15 min. Plasma was aliquot immediately stored at -80°C.

### Depletion of highly abundant proteins

Three different fractions of plasma samples were randomly pooled from each group, with each fraction containing approximately 5ml of pooled plasma from 10 individuals. Thus, there were 9 samples in total (Figure 7). The ProtoMiner protein enrichment kit (Bio-Rad, Hercules, CA, USA) was used in accordance with the manufacturer’s instructions. Total protein was determined by the Bradford method and protein normalization was confirmed using 12% SDS-PAGE.

### Protein digestion and iTRAQ labeling

Processed proteins (100μg) from each sample solution were digested with Trypsin Gold (Promega, USA) at 37°C for 12 hrs. iTRAQ proteomics experiments were performed in triplicates on pooled samples from 113- HH group, 115- control group, and 119- HH- related AIS group in accordance with the manufacturer’s instructions (iTRAQ regent kit; Applied Biosystems, USA) (Figure 7).

### Strong cation exchange fractionation and LC-MS/MS analysis

Chromatographic separation of pooled samples was performed on a LC-20AB HPLC system (Shimadzu, Japan). Eluted peptides were collected into 20 fractions. Supernatant peptides were loaded onto a LC-20AD nanoHPLC (Shimadzu, Japan) using an autosampler and a 2cm C18 trap column. Then, peptides were eluted onto a 15cm analytical C18 column, which was packed in-house. The injection volume was 10μl, and the flow rate was 300nl/min. Peptide acquisition was performed with a Triple TOF 5600 System (AB Sciex, USA) fitted with a Nanospray III source (AB Sciex) and a pulled quartz tip as the emitter (New Objectives, USA). MS/MS scans were performed in high sensitivity mode, as described previously [50].

### Protein identification and quantification

The ProteoWizard, tool msConvert, was used to convert MS/MS data into MGF format, and the exported MGF files were searched using MASCOT software version 2.3.02 (Matrix Science, UK) against the homos database (20184 sequences) downloaded from NCBI. For protein identification, the peptide mass tolerance was 0.05 Da, and the fragment mass tolerance was 0.1 Da; the peptide confidence cut-off was a Mascot Percolator Q-value <0.01, and each confident protein identification involved at least one unique peptide. For protein quantification, the IQuant software was used with a “picked protein false discovery rate” strategy [51]. To reduce false positive expression changes, DEPs should match with the following criteria: the protein must have been identified in at least 2 iTRAQ preparations, with at least two ratios >1.2 or <0.83-fold change.

### ELISA arrays

The plasma levels of AT-3, CRP, ApoB, and AHSG were detected using Homo ELISA kits (CUSABIO, China), as described by the manufacturer. A plate reader (Perlong Medical, China) with spectrophotometry at 450 nm was used to determine the absorbance of standards and samples and results were plotted against the linear portion of a standard curve.

### Statistical analysis

Statistical analysis of clinical characteristics and ELISA results was performed using SPSS 20.0 software (IBM, Chicago, IL, USA). Quantitative data were reported as means ± standard deviation, median and number (%), properly. Statistically significant differences between the three groups were calculated by t-test or chi-square analysis. Two-tailed p < 0.05 was considered to be statistically significant. Receiver operating characteristic curve (ROC) and the area under the curve (AUC) were used to assess the performance of proteins of interest to discriminate between the HH group and the HH-related AIS group.

## SUPPLEMENTARY MATERIALS FIGURES


