# Supplementary material for: Plasma proteomics reveals coagulation, inflammation, and metabolic shifts in H-type hypertension patients with and without acute ischemic stroke
Source: Oncotarget. 2017 Nov 1;8(59):100384–95. doi: 10.18632/oncotarget.22233 (PMC5725028; doi:10.18632/oncotarget.22233)
Supplement: Supplementary file 1 [file oncotarget-08-100384-s001.pdf]

Plasma proteomics reveals coagulation, inflammation, and metabolic shifts in H-type hypertension patients with and without acute ischemic stroke

SUPPLEMENTARY MATERIALS

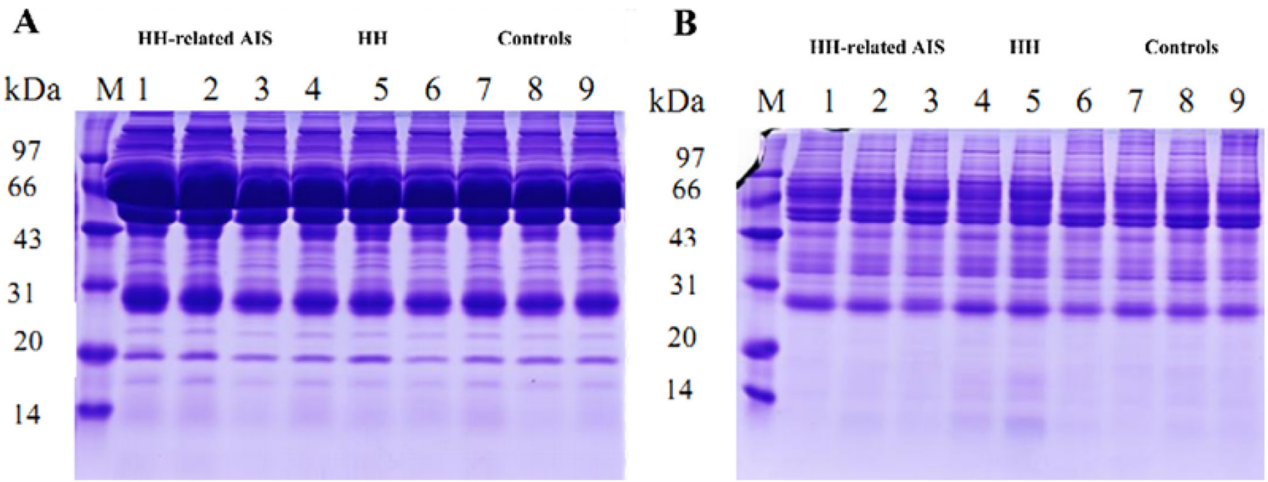

Supplementary Figure 1: SDS-PAGE profile of plasma samples from HH-related AIS patients, HH patients, and healthy controls. Sample lanes, each containing 30 µg of total protein, have been equally divided into 9 slices following tryptic in-gel digestion. Before **(A)** and after **(B)** depletion of highly abundant proteins.

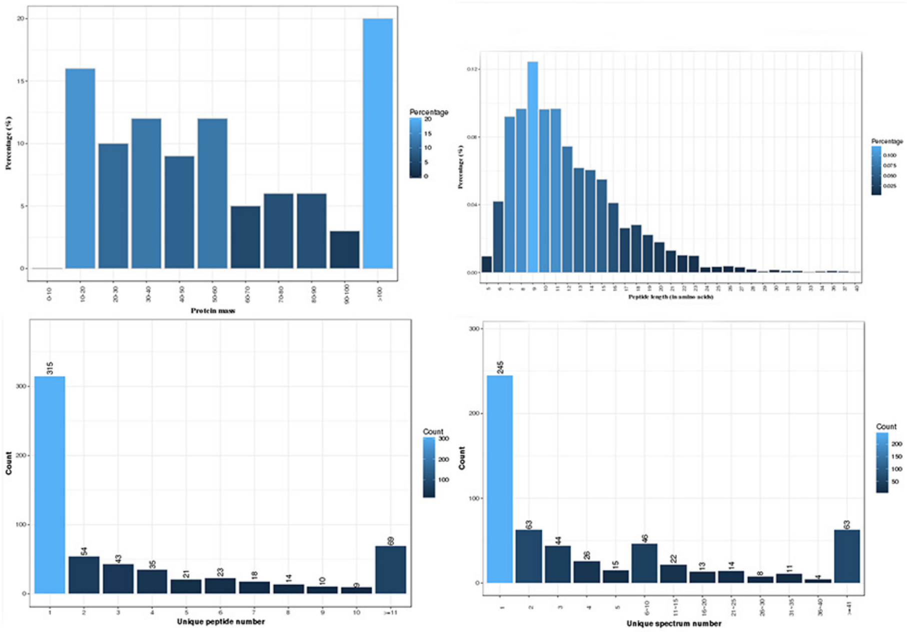

Supplementary Figure 2: Peptides identify and quantity. Protein mass **(A)**; peptide length **(B)**; unique peptide number **(C)**; unique spectrum number **(D)**.
